# Developing a Typology of Women's Attitudes Towards AI Use in the BreastScreen Programme—A Qualitative Study With BreastScreen Victoria Clients

**DOI:** 10.1111/hex.70415

**Published:** 2025-08-30

**Authors:** Maho Omori, Prabhathi Basnayake, Louise Keogh, Helen M. L. Frazer, Katrina Kunicki, Jocelyn F. Lippey

**Affiliations:** ^1^ BreastScreen Victoria Melbourne Victoria Australia; ^2^ Melbourne School of Population and Global Health University of Melbourne Melbourne Victoria Australia; ^3^ Torrens University Melbourne Victoria Australia; ^4^ St Vincent's BreastScreen Melbourne Victoria Australia; ^5^ Department of Radiology, Melbourne Medical School University of Melbourne Melbourne Victoria Australia; ^6^ St Vincent's Institute of Medical Research Melbourne Victoria Australia; ^7^ Department of Surgery, Melbourne Medical School University of Melbourne Melbourne Victoria Australia

**Keywords:** an AI attitude typology, artificial intelligence (AI), breast cancer screening, qualitative research, women's attitudes

## Abstract

**Background:**

There is growing scientific evidence supporting the potential of artificial intelligence (AI) to enhance breast cancer screening by improving the accuracy and efficiency of mammography interpretation. Aligned with this, several empirical studies, predominantly quantitative, have explored lay women's perceptions of AI in breast screening, often framing attitudes in binary terms—positive or negative. This approach can overlook the complexity and nuance of women's views.

**Aim:**

This article aims to unpack that complexity by developing a typology of women's attitudes towards the use of AI in the breast screening service. It builds on Birkland's (2019) information and communication technology (ICT) user typology among older adults and further explores the relationship between the attitude types and varying levels of AI acceptability.

**Method:**

Adopting an interpretative qualitative research approach, we conducted a combination of focus groups, paired interviews and one‐on‐one interviews with 26 women who had participated in the BreastScreen programme in Victoria, Australia. Data were thematically analysed using inductive coding.

**Findings:**

The analysis identified four attitude types—Enthusiast, Practicalist, Traditionalist and Guardian. Each type reflected unique motivations and experiences that shaped each participant's acceptance and rejection of AI. Most participants were classified as either Enthusiasts or Practicalists, indicating a generally high or moderate level of AI acceptance. Enthusiasts viewed AI as an exciting and necessary progression, and Practicalists valued its practical utility as a useful tool. Both groups shared the belief that AI represents the future of healthcare, underpinned by technological advancement. Traditionalists, on the other hand, expressed a preference for the status quo, advocating for the exclusive role of human doctors. Guardians typically had higher levels of AI knowledge and advocated for a cautious approach, citing social and ethical concerns about AI integration.

**Conclusion:**

The typology illustrates that the BreastScreen Victoria clients' attitudes towards AI are more nuanced and dynamic than a simple positive–negative dichotomy. Recognising these perspectives is critical for designing AI implementation strategies that are sensitive to the needs and concerns of care recipients.

**Patient or Public Contribution:**

This study was shaped by extensive stakeholder engagement with BreastScreen Victoria and its consumer representatives from the outset. Research materials were collaboratively developed and reviewed, ensuring the study design was fit‐for‐purpose.

## 1 Introduction/Background

With the rapid advancement of artificial intelligence (AI), there has been significant attention on its potential to transform the existing healthcare services. Radiology is a natural focal point for AI development given its pre‐existing digital nature, large well well‐curated data sets and rigorous quality assurance. Currently, more than 700 AI‐operated medical devices in radiology have received FDA approval, including over 20 in breast imaging [1]. Research continues to demonstrate the efficacy and effectiveness of AI‐operated applications across various medical fields [2, 3, 4].

As medical AI advances, research into public views towards AI in healthcare settings has grown. A global survey with a total of 13,806 patients across 74 hospitals in 43 countries has found that more than half of the respondents supported the general use of AI in healthcare and AI assistance in medical diagnosis [5]. Attitudes varied by demographics—older male patients in poorer health and female patients were less supportive of AI than younger, tech‐savvy male patients [6]. Another large‐scale survey supports the demographic influence on AI attitudes but found less than half of the survey respondents supported the use of AI in diagnosis specifically [7]. Khullar et al. report overall optimism with most survey participants believing AI would improve healthcare, though their comfort levels depended on clinical applications, for example, they were more comfortable with AI reading chest x‐rays than diagnosing cancer [8].

Other quantitative and qualitative studies have explored care recipients' views on AI in specific clinical settings, including CT, MRI and conventional radiography, cardiac imaging, neurosurgery, mental health and echocardiogram [6, 8, 9, 10, 11, 12]. Across these contexts, participants consistently showed greater acceptability when AI was used collaboratively with doctors. Conversely, participants expressed discomfort with AI operating autonomously such as interpretating diagnostics or delivering care without doctors' oversight. These findings suggest public acceptability of AI is contingent on their view that AI serves as a supportive tool rather than an independent decision‐maker.

### 1.1 Women's Attitudes to AI Use in Interpreting Breast Screening Mammography

Recent studies, primarily quantitative, from the United Kingdom, Sweden, Norway, the Netherlands, Italy and Australia have examined women's^1^ attitudes towards AI use in breast screening. These studies find general support for AI integration into breast screening services, though demographic factors influenced their acceptance levels [13, 14, 15, 16, 17, 18, 19]. Higher AI literacy and education were associated with more supportive views [16, 17]. However, one study found that women over 50, who are more likely to have undergone screening, supported AI more than younger cohorts with no screening experience, despite having lower familiarity with AI or AI‐based health applications [15].

Some differences emerged across the studies. The Swedish study found strong support for both fully autonomous and partial AI‐assisted decision‐making, with the highest level of trust placed in doctor–AI collaboration in mammogram reading [14]. In contrast, studies from the United Kingdom, the Netherlands and Italy reported lower support for AI operating independently [16, 17, 18], suggesting that women's positive attitudes are conditional and potentially influenced by cultural context. Three qualitative studies from Australia, the United Kingdom and Sweden offer deeper insights into the quantitative findings [13, 15, 19]. Focus group participants showed generally positive views, highlighting potential benefits, such as improved accuracy, efficiency and health outcomes when AI is used alongside radiologists [13, 15, 19]. They also emphasised the importance of fairness and inclusiveness, particularly minimising discriminatory biases, and the need for thorough AI evaluation, data privacy protections and clear accountability in the event of errors [13, 15, 19].

### 1.2 This Study

While women generally express positive but conditional support for AI use in breast screening, existing studies mainly focus on demographics and perceived benefits and risks, overlooking deeper motivations. This article addresses this gap by exploring the underlying dynamics behind both ‘positive′ and ‘negative′ attitudes towards AI. Moving beyond these binary classifications, we develop an AI attitude typology that captures how personal experiences and health values shape women's views on AI in breast screening.

To inform our approach, we drew on literature from Gerontechnology research that explores how technology supports or enhances older people's quality of life [20]. In particular, we were inspired by Birkland's information and communication technology (ICT) user typology based on her extensive qualitative study [21]. The typology offers valuable insights into the factors influencing older individuals' acceptance, adoption and rejection of technologies. Her framework identifies five user types, four of which—Enthusiast, Practicalist, Traditionalist and Guardian—are particularly relevant to understanding women's orientations towards AI use in breast screening (Table 1). This typology helped shape our data analysis, which is detailed in the methodology section.

**Table 1:** Birkland's (information and communication technology) ICT user typology.

Enthusiast	ICTs are fundamental part of enthusiasts' lives. They view technology as an enjoyable tool and are eager to incorporate it into every aspect of their daily lives. An inability to use these technologies negatively impacts their overall life satisfaction.
Practicalist	For practicalists, the use of ICTs is task‐driven, with the primary goal of accomplishing a specific task. They do not necessarily enjoy being constantly surrounded by them in their daily lives.
Traditionalist	Traditionalists have strong affection for the technologies of their youth, such as TV and radio. They have the lowest ICT skills among all user types and hardly go online.
Guardian	Guardians have general distrust of all technologies and strictly regulate their use. They foresee potential risks to society when ICTs are used in an uncontrolled manner.

## 2 Methodology

This study adopts an interpretive qualitative research approach, grouped under the grounded theory tradition, based on the premise that social actors construct meaning through a ‘sense‐making′ process within their social environments [22]. The approach is suited to exploratory research, allowing researchers to understand dynamic social processes within which people's perspectives unfold [22]. It helped the research team gain understanding of how women's attitudes towards AI are influenced by their lived experiences. To ensure the robustness and transparency of our findings, we report this qualitative study in align with the Consolidated Criteria for Reporting Qualitative Research (COREQ) checklist [23]. This study received ethics approval from the Human Research Ethics Committee of University of Melbourne.

The study was conducted in the context of BreastScreen Victoria, Australia's government‐funded, population‐based breast cancer screening programme. It offers biennial 2D digital mammograms to asymptomatic women aged 50–74 with optional participation for those aged 40–49 and those over 74. There are 55 screening sites across Victoria, and mammograms taken at local screening sites are sent to the eight central Reading and Assessment Services (RASs), where two radiologists independently review the images. Discrepancies are resolved by a third senior arbitration radiologist. Further assessment is provided for those who are recalled with abnormal mammogram findings. Consumer representatives from BreastScreen Victoria contributed to the study's development, including focus group questions and recruitment materials.

### 2.1 Recruitment and Data Collection

Data collection occurred between February and September 2022. Recruitment targeted existing BreastScreen Victoria clients—women who have previously participated in the BreastScreen Victoria programme. It occurred in two phases. In the first phase, a random sample of BreastScreen Victoria clients were approached via email and invited to voluntarily participate in online focus groups (via Zoom) due to local COVID restrictions at the time. However, those participated in the first phase were demographically homogenous, predominantly Anglo‐white women, aged 60–70 with similar educational backgrounds. This prompted a more targeted second phase.

In the second phase, 200 paper invitation with the plain language statement and consent form were distributed at five selected screening sites to potential participants, either before or during their screening appointments. Interested participants returned signed consent forms during their visit, providing their contact details for follow‐up. Data collection during the second phase was conducted in person with the COVID restriction lifted.

Although focus groups were the preferred for this exploratory study, a combination of focus groups, one‐on‐one, and paired interviews was conducted to accommodate participants' availability. All sessions were recorded with participants' consent and transcribed verbatim. Semi‐structured, open‐ended questions encouraged participant‐led exploration and minimised researchers' bias. Sessions began with participants' general views on AI in everyday life and in broader healthcare contexts. Following this, a brief explanation of the current mammogram reading and the potential role of AI within the BreastScreen Victoria programme was provided. Participants were then asked about their feelings towards AI use in a mammogram reading. The focus group (interview) guide is available as Supporting Information.

Three authors (P.B., J.F.L. and K.K.) facilitated the sessions. None were previously known to participants. One facilitator was directly involved in the BreastScreen programme as a practising surgeon when data collection occurred, whereas the other two had no direct involvement in the programme. After each session, the research team debriefed to review key insights. As data collection progressed, the team assessed whether new insights were still emerging, ensuring that data collection continued until no new themes emerged. The final two paired interviews revealed no new insights, confirming data saturation was reached.

### 2.2 Data Analysis

Thematic analysis was guided by an interpretive approach. After completing data collection and transcription, three researchers independently reviewed the transcripts to build familiarity, sharing field and reflection notes. Open coding was performed to identify patterns within the data. Once various patterns (codes) were identified, we compared them, seeking similarities, shared meanings, differences and potential causal relationships. Related codes were grouped into overarching themes. Research rigor was ensured through a systematic, transparent and independent coding process, which led to shared interpretations among the team.

Data analysis revealed distinct attitude types towards AI use in the BreastScreen programme, spanning a spectrum from positive to negative, the nuances of which were not previously documented in the literature on public views of healthcare AI. To contextualise these findings, we undertook a comprehensive literature review to search existing, empirically validated conceptual models and found Birkland's ICT user typology as a particularly relevant framework [21]. This framework, which explores older people's technology adoption through a lens of reflexivity, helped us align and name our emergent attitude types. We further refined our typology by exploring additional characteristics specific to each attitude category.

### 2.3 Participants

Two focus groups, six paired interviews and four one‐on‐one interviews were conducted with a total of 26 BreastScreen Victoria clients. The participant's age, duration of receiving the services and final educational attainment varied (Table 2). The interviews lasted between 35 and 50 min, whereas the focus groups lasted about 70 min. To maintain anonymity, all participants are assigned pseudonyms. In the finding section, the numerical values following the pseudonyms represent the participants' ages.

**Table 2:** Participants' characteristics.

*Age*	
> 50	0 (0%)
50–59	5 (19%)
60–69	5 (19%)
70–79	15(58%)
> 80	1 (4%)
*Experience with BreastScreen *	
< 5 years	3 (12%)
5–10 years	5 (19%)
11–20 years	3 (12%)
> 20 years	10 (38%)
Unknown	5 (19%)
*Education qualification*	
High school	7 (27%)
Diploma/certificate	12 (46%)
University degree or postgraduate	7 (27%)

## 3 Findings

**Figure 1:**
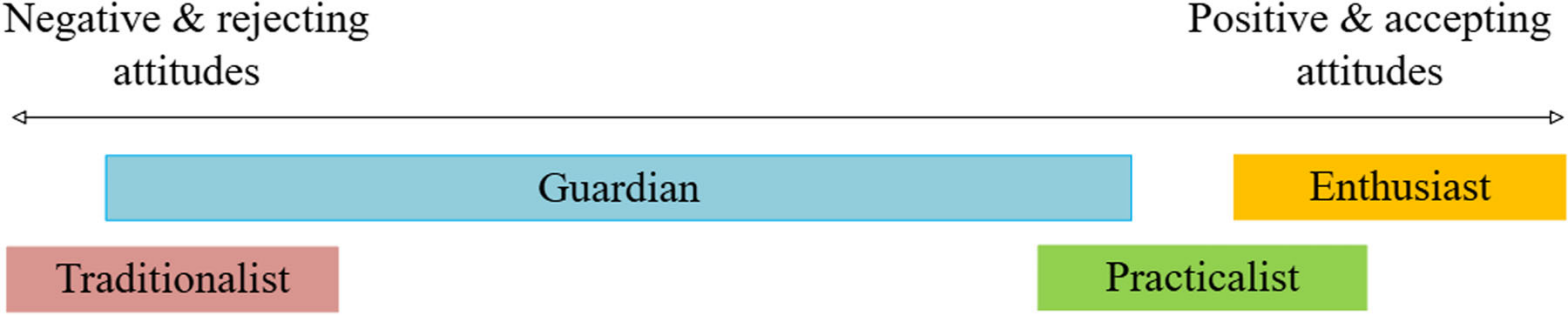
Attitude types under the ‘negative’ and ‘positive’ spectrum.

Focus groups and face‐to‐face interviews revealed a range of nuanced views on AI in the BreastScreen programme. Many participants echoed findings from the previous studies, highlighting potential advantages and perceived benefits as well as concerns about the potential for errors and accountability for machine mistakes. However, they did not adopt simple ‘positive′ or ‘negative′ positions but instead, expressed more nuanced and complex, experience‐based perspectives. Using Birkland's the ICT users typology, we developed a spectrum‐based typology of attitudes towards AI, illustrated in Figure 1. While the Enthusiast, Practicalist and Traditionalist represent consistent stance—either positive or negative—the Guardian spans both ends of the spectrum, shown in the longer block representing this group. Each type is described in Table 3.

**Table 3:** A typology of women's attitudes to AI in BreastScreen and brief description of each attitude type.

Attitude type	Positive or negative	Description	Participants (name [pseudonym] and age)
Enthusiasts (*n* = 13)	Positive	Strong enthusiasm about AI being used in the programme Belief that AI is the way to forward Solid knowledge and understanding of AI Lived experience with the benefits of AI–doctor collaboration Lived experience with effective AI–human collaboration in the workplace High level of trust in AI	Dina (69), Pat (74), Kim (74), Terry (71), Judith (71), Connie (71), Lynne (70), Kath (70), Pam (72), Meg (55), Jo (72), Sue (69), Deb (74)
Practicalists (*n* = 8)	Positive	Hold positive views of AI and recognise its potential for improving practice View AI as a useful tool with practical value Based on more intuition than extensive knowledge No lived experience with AI‐operated healthcare services or working with AI in the workplace	Michelle (50), Margaret (68), Sharon (73), Chris (58), Vicki (54), Karen (66), Yvonne (81), Lori (72)
Traditionalists (*n* = 2)	Negative	Little understanding of technology or no desire to acquire tech knowledge Prefer human doctors in all circumstances Feeling that introducing AI in the BreastScreen programme would be a concession	Julie (69), Liz (74)
Guardians (*n* = 3)	Either positive or negative, or both positive and negative	Concerned about data security and privacy Capable of understanding AI (not necessarily opposed to it) Interested in scientific evidence supporting AI use Ability to foresee potential risks or catastrophes caused by AI	Mel (54), Martha (71), Barbara (70)

**Enthusiasts**—I'm all for advancement, and this will improve the ability to read [mammograms], I'm all for it with my mammogram experience.

(Pam, 72)

Enthusiasts viewed the introduction of AI in the BreastScreen programme as a natural step forward, especially given the growing presence of AI‐operated applications in our daily lives. The 13 participants in this category were excited about the idea of incorporating AI into the programme, believing that it would significantly benefit. Dina (69) expressed, ‘I'm a fan [of AI]. I think it's going to make a dramatic difference, the speed and accuracy [of reading mammograms]. … in terms of anything that requires mass screening, it's got to make a big difference, be more efficient, and release resources for more difficult pieces’.

Not all enthusiasts were computer literate or familiar with advanced technology in their everyday lives. For example, Kim (74) described herself as computer illiterate, saying ‘I'm jealous actually when I see people go to the computer and just go “click, click, click…” I wish I could do that’. Despite this, she expressed strong support for AI in the breast screening services, saying, ‘The whole idea is to find if there's a problem [in a mammogram]. So, if two heads are better than one, whether it's two humans or a human and a computer, it doesn't really matter to me as long as they get an answer’.

The strong ‘pro‐AI’ stance held by the enthusiasts was rooted in their unique lived experiences. Judith (71), a breast cancer survivor, shared her experience of cancer diagnosis by her doctor within a year after receiving an ‘all clear’ result from the BreastScreen programme. She was unsure whether her cancer had been missed or simply was not visible in the mammograms at the time. Judith reflected, ‘With my experience, I'd probably prefer that (AI) as well as a radiologist analysing the results. I would have liked to have gone back two years and had artificial intelligence read my mammogram to see if there was anything they could have picked up’. Judith's desire to improve the current system with AI was echoed by Kath (70) and Pam (72), both of whom had been called back for further testing after their initial mammogram results. Although no cancer was detected, the process of undergoing extensive assessments was stressful and anxiety‐inducing for both. Kath (70) described her experience:

I had a call back … it (the assessment) was very thorough, … it took a really long time because the tests had to be done over and over, because they (doctors) couldn't see what they were looking for… I think if AI could help the diagnosis, it'd be easier and less stressful. Maybe a computer would see it more clearly, and I wouldn't have been there.

Enthusiasm for some participants was influenced by positive experiences with AI or machine‐assisted treatments in other contexts. Terry (71) had a positive experience of undergoing robotic knee surgery, whereas Jo (72) received machine‐assisted care for a broken wrist. Connie (71) shared that her husband's life was saved by an AI‐operated defibrillator fitted in his chest. Sue (69) had her thyroid condition identified through a machine and explained:

I'm quite positive about AI. Recently I went in for some minor surgery and had a MRI, which revealed a lump or something in the neck area. It turned out I had nodules on my thyroid. I was really pleased that interpretation happened. The doctors could see there was swelling, but they didn't know what was causing that swelling, whereas a deeper look with the machine could find out what was going on underneath. So, I'm quite confident.

Positive experience working with AI made some to believe in its potential, even in the context of breast screening. Meg (55), who works in the government agency where AI‐operated systems are extensively used noted that it has significantly enhanced the quality and efficiency of human work. She shared her perspective on AI in breast screening:

I think anything that can help screen better, to pick up things earlier, would be good. As a human, we are not infallible. When you're using our interpretation to analyse something, sometimes we get it terribly wrong. Whereas, when you put parameters into a computer, it just works with what's in front of it, and assess it all. I might have a mammogram, and AI might pick up something that a radiologist doesn't. So, AI is, I guess, an additional layer of assessment.

Meg and others emphasised that AI–human collaboration was key to achieve outcomes superior to human performance alone.

The common trend among the enthusiasts was the belief that integrating AI into the BreastScreen programme would enhance their trust and confidence in the service. Terry (71) expressed, ‘Would I have more faith [in the BreastScreen program]? I think yes. I just wonder if all those mammograms are fed into the machine, the machine ends up being more reliable’.

**Practicalists**—It (AI) is a good tool to add to the doctor's toolbox box.

(Michelle, 50)

In Birkland′s work, Practicalists view technology as a functional tool, rather than something to be excited about. This pattern was found among eight participants. These individuals expressed acceptance of its use as a means of enhancing the existing practices. Unlike the Enthusiasts, they show little excitement about new technology or innovation in healthcare. Some even considered adjusting their behaviour if AI were to positively impact the programme. Sharon (73) expressed,

If it's (AI is) going to improve things, find [more cancer] or help people, bring it on. If it′s going to be a change, I'll have it. Things are changing, it (the AI product) wouldn't be out there on the market if the artificial intelligence wasn't of the highest quality and doing its job. If that was going to get my breast screen done, I'd be a little hesitant, but I'd get to change my ways.

Practicalists had no direct experience with AI in healthcare, either personally or through family, friends or work, and therefore, formed their view without firsthand experiences or exposure. Their acceptance of AI was largely driven by the perception that this is the direction society is heading. Their levels of understanding of AI varied; some knew more, and some knew little. During focus group discussions, some participants initially had little to no understanding but gradually developed ideas about its potential benefits and risks in the BreastScreen context, ultimately recognising its value. Yvonne (81), for example, initially said ‘I'd rather have the human [in the services]’ but by the end of the focus group, she changed her mind, saying ‘You've swayed me now. I would say yes, I'd go with the computer, no problem at all’.

Some became more favourable for AI after discussions about its objectivity. Karen (66) explained, ‘They (machines) never get sick, they don't have reasons to have a day off, they don't have bad days… if it's in our best interest that computers do it because they don't make mistakes, then what's the problem of using it?’

However, this objectivity was contested by Michelle (50) who had higher AI literacy through her family influence. While she acknowledges the recent advancements in AI, her understanding made her more cautious, stating:

There's a lot of potential there, but also you need to be careful with how you set things up. For me I think it's important to make sure that you have human input alongside AI. I think in medicine, it's not just about the facts and figures… Sometimes it may be a little about instinct and having a human component where you can rely on your [instinct].

Like Michelle, some believed objectivity alone is not sufficient due to the absence of human elements. They emphasised the importance of AI–human collaboration and viewed AI as an assistive tool that complements doctors.

**Traditionalists**—I still prefer to have the human (radiologist).

(Liz, 74)

Reflecting Birkland′s definition, traditionalists in our study preferred familiar, conventional, doctor‐led healthcare service delivery over technological intervention. Two participants fit this type. Liz (74) is a technology sceptic (or technophobic), stating ‘I'm not into technical stuff at all, I don't want to know about it. I get TV or whatever at home, I get my husband [for help] and say, “you click”’. Throughout the focus group, her views on AI were not influenced by her fellow participants. She remarked, ‘It seems like everything is going that way, I'd prefer a person, but I'd just given in’.

Unlike Liz, Julie (69) was not entirely opposed to technology, but felt uncomfortable with the idea of accepting AI in healthcare and breast screening. Her traditionalist stance stemmed from a sense of uncertainty than a dislike of technology. After hearing about her fellow participant's positive experience with the robotic knee surgery, she expressed her discomfort: ‘I feel a bit uncomfortable about that… because human bodies react differently and how's all that going to be included in the algorithm?’ When discussion turned to AI in breast screening, she noted,

I don't understand enough about the technology to have the confidence in it. I think probably more explanation would persuade me… I guess part of it is the way society's heading though. Is everybody comfortable with the fact that more things are being run by machine and computers, and human elements are taken out? I don't like it.

Although Julie remained resistant to AI in BreastScreen programme during the interview, her traditional stance might shift with more detailed explanations.

Guardians—It's (AI is) more about invasion of privacy.

(Martha, 71)

Guardians as defined by Birkland, were concerned about technology's broader societal impact, particularly around data security and privacy. Three participants fit this type. They were neither technology sceptics nor outright opposed to AI, but they were aware of the risks and dangers associated with its use. The Guardians had different personal experiences that shaped their viewpoints.

Mel (54) who had worked in the IT industry, possessed a solid technical understanding of AI, including its capability and how it is designed and developed. Her cautious stance was rooted in this knowledge. She expressed concerns about privacy, stating:

I would have privacy concerns because you don't really know where your data is, and anyone can hack into any kind of database, I'm quite sure. I don't know how safe it is, but I don't think there's any other way these days to keep your information [safe], in terms of privacy. But we wouldn't go back to the days when everything was on file. …I feel this is a bit scary, but it is also good.

Mel acknowledged both the potential benefits of AI, such as its superior ability to recognise patterns, and the risks associated with its use in healthcare. At the same time, she emphasised the necessity of improving the healthcare system with technology. Her comments align with Birkland′s description of guardians, who are willing to cautiously accept technology, ‘if they are convinced that it is secure′ (p. 77).

In contrast, Barbara (70) had a different perspective. Although she was familiar with AI, her understanding was not as technical as Mel's. Barbara's opposition to AI was driven by her scepticism towards corporate greed, concern about motivations and distrust in the government. Early in the interview, she asked, ‘Who is pushing this (AI advancement in the BreastScreen program)? Is it a company that's going to benefit? My concern is, is it for our benefit or for business?' After the interviewer explained that AI advancement was motivated by addressing the chronic shortages of radiologists, improving accuracy and enhancing efficiency in mammogram reading, without involvement of profit‐driven entities, Barbara softened her stance. However, when the discussion turned to privacy and data security, she became even more opposed, stating, ‘They (BreastScreen) know far too much information about us… I just don't trust where that information goes. …I have no trust in the system.’ Barbara emphasised that her opposition to AI would not change unless the government became more diligent about protecting individuals' privacy.

## 4 Discussion

This study contributes to the growing body of literature on public attitudes towards AI in healthcare by offering deeper insights into women's perspectives within the context of breast screening. Using the typology informed by Birkland [21], it reveals how BreastScreen Victoria clients form context‐dependent yet conditional views on AI that go beyond simply positive or negative oppositional stances. The nuanced attitudes, shaped by lived experiences, extend beyond what the demographic factors typically capture in survey‐based research. The focus groups and interviews functioned as a reflective space where participants actively explored and developed their views through dialogue.

Reflexivity, a key concept in social sciences, refers to how individuals in the modern society constantly process information to adjust, adapt and correct behaviour through ongoing reflections. Giddens introduces the term ‘reflexive modernisation′ [24], arguing that individuals are required to ‘make sense of rapid changes and make personal decisions based on some form of risk assessment′ [25]. Reflexivity not only fosters a positive outlook but can also generate a sense of uncertainty [26]. By examining potential benefits and risks of integrating AI in the BreastScreen programme through the lens of lived experiences, participants developed varied and dynamic views towards the novel technology in healthcare services. They were categorised into four attitude types—Enthusiasts, Practicalists, Traditionalists and Guardians.

Most participants held positive attitudes towards AI use in the BreastScreen programme, consistent with previous studies [13, 14, 15, 16, 17, 18]. This optimism took two forms: Enthusiasts and Practicalists, both of whom saw ‘AI is the way forward′, given the recent advancement in technology. However, the reasoning differed among these two groups. Participants fell in the Traditionalist and Guardian categories expressed negative attitudes towards AI. While the Traditionalists resisted change, the Guardians were more informed and cautious shaped by awareness of AI's broader impact. This study reveals that strong support for AI does not always stem from technical familiarity. Some Enthusiasts openly acknowledged their limited technical skills and knowledge, but remained optimistic about AI's potential to improve the accuracy of mammogram reading. Their support was often influenced by own lived experiences with breast cancer diagnosis or medical procedures in other healthcare contexts, highlighting the significance of experiential knowledge in shaping ones' openness to innovation. This may help explain findings of Lennox‐Chhugani et al. [15], which show that women over 50 (the target age group for screening) express stronger support for the use of AI in breast screening, despite having lower technology literacy compared to younger women. These findings challenge common assumptions that AI acceptance is driven mainly by younger age, higher education and higher technology literacy [5, 27].

A concept of ‘AI–human collaboration’ is present across all attitude types, though interpreted differently. Overall, AI was seen as a transformative tool to augment, not replace human expertise [28, 29]. Many Enthusiasts and some Guardians saw AI as potentially superior to human doctors, believing it could detect details that human eyes might miss. Therefore, they considered AI–doctors collaboration would enhance overall service quality. In contrast, Practicalists valued AI's objectivity, however, emphasised its limitation as a machine—the lack of tacit knowledge [30]—meaning that doctors and AI could build a unique partnership [29]. For the Guardians, human involvement remains essential to regulate the system, ensuring the safe and ethical implementation of AI.

A sense of trust varied across attitude types. Enthusiasts were confident in AI's potential and believed its integration into the BreastScreen programme would strengthen their trust in the service. While many Practicalists rarely mentioned trust explicitly, their comments often implied it like Sharon′s remark that AI would not be on the market without meeting a high standard, reflecting underpinning confidence in technology. In contrast, Traditionalists expressed no trust in AI and instead expressed trust only in human doctors. For Guardians, trust is centred on ethical concerns, particularly transparency in handling personal health data. While previous research discusses that trust is key to AI acceptability [31], our findings extend this by showing that trust in a broader healthcare system is equally important to trust in the technology itself.

Among the Practicalists, the acceptability of AI was not fixed. Many of them began the focus groups and interviews with little to no experiential knowledge about AI and expressed uncertainty about its use. Their views evolved through discussions, particularly hearing others' experiences, gaining a clearer understanding of AI's capabilities and recognising its supportive role, rather than replacing human doctors. This shows the significant influence of meaningful engagement and accessible information in shaping ones' attitudes. It implies the importance of public engagement, participatory dialogues and the potential role of education tools for the successful implementation of AI in healthcare.

While the study findings were exclusively drawn from the breast screening context, the attitudinal typology identified in this study could have a broader application across other healthcare domains given that the underlying perceptions and concerns are not unique to breast screening. Further research may be needed to explore if similar attitudinal patterns exist in different clinical settings within and beyond Australia, which would help validate the applicability of our typology.

### 4.1 Limitations of This Study

There are some limitations to consider. First, there is a noticeable gap between the data collection period and the reporting period. Given the rapid evolution of healthcare AI and its real‐world applications, the data may be somewhat dated. However, growing research into public attitudes towards AI across various healthcare domains has reached similar findings, likely reflecting the slower AI adoptions in clinical settings. Importantly, none of the existing research has examined the nuanced and varying dynamics of these attitudes by developing an attitudinal typology. Therefore, we argue that our data remains valuable in contributing to the development of new knowledge. Second, the participants were not wholly representative of BreastScreen Victoria clients as we relied on their voluntary participation, meaning that they were self‐selected and expressed interest in joining our discussion sessions, which may have introduced a degree of bias. However, this limitation is consistent with the nature of qualitative research, which does not aim to generalise findings [22]. Instead, our objective was to explore patterns or tendencies within a specific group, providing depth and context to their experiences and perspectives. While generalisation is beyond the scope of this study, future research could adopt a mix‐methods approach to enable triangulation and enhance the breadth and applicability of findings.

## 5 Conclusion

The typology of Enthusiasts, Practicalists, Traditionalists and Guardians provides a nuanced understanding of consumer support for and resistance to AI in the BreastScreen programme. Reflexivity played a key role in shaping these attitudes, drawing on personal experiences and engaging in external dialogue. These attitudes are not fixed but malleable, influenced by how AI is communicated and the type of information provided to lay individuals. Given the variation in participants' knowledge, interests and concerns about AI, tailored communication is essential to address the distinct needs of each attitude type. While this study specifically focused on breast screening, the typology may be applicable to other emotion‐sensitive healthcare settings across different cultural contexts. Future research should explore whether similar attitude patterns emerge in different healthcare domains, and how these attitudes evolve following the real‐world implementation of AI.

## Author Contributions

Conceptualisation: M.O., P.B., and J.F.L. Methodology: M.O., P.B., and J.F.L. Investigation: P.B., J.F.L., and K.K. Formal analysis: M.O., P.B., and J.F.L. Writing – Original Draft: M.O., P.B., and J.F.L. Writing – Review and Editing: L.K., H.M.L.F., and K.K. Viaualization: M.O. Supervision: L.K. and J.F.L. Project administration: K.K. Funding acquisition: H.M.L.F. and J.F.L.

## Ethics Statement

This study was approved by the Human Research Ethics Committee of University of Melbourne (Ref no: 2021‐22029‐20158‐4). All participants provided their signed the consent forms before or at the time of participating in the focus groups and interviews.

## Conflicts of Interest

The authors declare no conflicts of interest.

## Supporting information

Client Focus Group Guide.

## Data Availability

Data (collated data) will be available upon request.

## Endnotes

1 Here the women include both recipients and non‐recipients of breast cancer screening service.
