# Supplementary material for: Developing a Typology of Women's Attitudes Towards AI Use in the BreastScreen Programme—A Qualitative Study With BreastScreen Victoria Clients
Source: Health Expect. 2025 Aug 30;28(5):e70415. doi: 10.1111/hex.70415 (PMC12397983; doi:10.1111/hex.70415)
Supplement: Supplementary file 1 — Client Focus Group Guide. [file HEX-28-e70415-s001.docx]

# Client Focus Group Guide

## Section 1- General Warmup

1. First, can you please tell us a bit about yourself, what you do and what interested you in expressing your interest in participating in this focus group?
2. What is your general experience in BreastScreen Victoria and how long have you been a client of BreastScreen?

For the purposes of this discussion, when we refer to artificial intelligence, we mean the use of machines or computers to do the interpreting, diagnostics or analysis of information that a human would do.

## Section 2- Perceptions of artificial intelligence (AI)

1. What is the first thing that comes to mind when you think of AI?

*Prompt- if you express it in one or two words, what would it be?*

*Can you please tell us your general view of AI?*

## Section 3- Experience of AI in health or other sectors

1. What are your general thoughts about using AI in health care?

*Prompt: General opinions about AI and their potential in services*

*Prompt: Have you had experience with AI being involved in your health care in any other setting?*

[Providing information about the current mammogram reading process, and potential use of AI in mammogram reading]

*Before we discuss using AI for mammograms, we will explain what happens in BreastScreen currently. After you have your mammogram it is looked at by 2 independent radiologists. If they disagree a third radiologist has a look at the mammogram and arbitrates if it is clear or needs more tests.*

Section 4 – AI in mammographic screening/reading

1. What do you think of involving AI being used in reading your mammograms?

(*Explain briefly and simply what this means- By involving AI we mean your results are generated and assessed by the help of an artificial intelligence/ algorithm/ model*)

*Prompt: “If positive” — Can you please elaborate?*

*“If negative” — Can you tell us why you think so?*

*Prompt: What do you think some advantages might be of AI to read your*  *mammograms?*

*What may be some disadvantages may be?*

*Prompt: Do you have concerns about AI interpreting your mammograms?*

1. How would you like to be informed AI is being used to read your mammograms?

*Prompt: When making the booking? As you register on the day of your mammogram? Via email?*

## Section 5- Accuracy, Privacy and Legal Concerns

1. What impact would having AI involved in interpreting your mammogram have in your confidence or trust in BreastScreen?

*Prompt: What would be your concerns about it?*

1. What feelings do you have about a radiologist making a mistake compared to a machine making a mistake in reading your mammogram?

## Section 6- Acceptability and Marketing aspects

1. How do you think other women within BreastScreen would accept the use of AI use in mammography?

*Prompt: What groups would find it easy/hard to accept the AI involvement in mammography?*

1. If AI is involved in screening in a safe way (preserving privacy etc), how would you want the fellow BSV clients to know about it first?

*Prompt: What are the best ways to inform others about BreastScreen Victoria’s AI involvement in mammography.*

1. What script do you think is best to give other clients? (*Alternative question)*
